# Ca^2+^ signaling in vascular smooth muscle and endothelial cells in blood vessel remodeling: a review

**DOI:** 10.1186/s41232-024-00363-0

**Published:** 2024-12-27

**Authors:** Yoshiaki Suzuki, Wayne R. Giles, Gerald W. Zamponi, Rubii Kondo, Yuji Imaizumi, Hisao Yamamura

**Affiliations:** 1 Department of Molecular and Cellular Pharmacology, Graduate School of Pharmaceutical Sciences, Nagoya City University, Nagoya, 467-8603 Japan; 2 Department of Physiology & Pharmacology, Cumming School of Medicine, University of Calgary, Calgary, AB T2N 4N1 Canada; 3 Department of Clinical Neurosciences, Hotchkiss Brain Institute, Alberta Children’s Hospital Research Institute, Cumming School of Medicine, University of Calgary, Calgary, AB T2N 4N1 Canada

**Keywords:** Ca^2+^ signaling, Endothelial cells, Hypertension, Macrophages, Monocytes, Vascular remodeling, Vascular smooth muscle cells

## Abstract

Vascular smooth muscle cells (VSMCs) and endothelial cells (ECs) act together to regulate blood pressure and systemic blood flow by appropriately adjusting blood vessel diameter in response to biochemical or biomechanical stimuli. Ion channels that are expressed in these cells regulate membrane potential and cytosolic Ca^2+^ concentration ([Ca^2+^]_cyt_) in response to such stimuli. The subsets of these ion channels involved in Ca^2+^ signaling often form molecular complexes with intracellular molecules via scaffolding proteins. This allows Ca^2+^ signaling to be tightly controlled in localized areas within the cell, resulting in a balanced vascular tone. When hypertensive stimuli are applied to blood vessels for extended periods, gene expression in these vascular cells can change dramatically. For example, alteration in ion channel expression often induces electrical remodeling that produces a depolarization of the membrane potential and elevated [Ca^2+^]_cyt_. Coupled with endothelial dysfunction blood vessels undergo functional remodeling characterized by enhanced vasoconstriction. In addition, pathological challenges to vascular cells can induce inflammatory gene products that may promote leukocyte infiltration, in part through Ca^2+^-dependent pathways. Macrophages accumulating in the vascular adventitia promote fibrosis through extracellular matrix turnover, and cause structural remodeling of blood vessels. This functional and structural remodeling often leads to chronic hypertension affecting not only blood vessels, but also multiple organs including the brain, kidneys, and heart, thus increasing the risk of severe cardiovascular events. In this review, we outline recent advances in multidisciplinary research concerning Ca^2+^ signaling in VSMCs and ECs, with an emphasis on the mechanisms underlying functional and structural vascular remodeling.

## Background

Arteries consist of a three-layer structure: the intima, media, and adventitia, with endothelial cells (ECs), vascular smooth muscle cells (VSMCs), and fibroblasts being the main component cells of each layer. The adventitia also includes resident immune cells and autonomic nerve endings. Arteries can be classified into three types on the basis of their vessel diameter and contractility patterns: elastic arteries, muscular arteries, and resistance arteries [1]. Elastic arteries are rich in elastic fibers and contribute to vascular compliance in response to cyclic high-pressure blood flow from the beating heart. In contrast, resistance arteries/arterioles can develop high contractile forces. Resistance arteries/arterioles dynamically change their diameter as a primary mechanism to regulate systemic blood pressure [2]. In healthy blood vessels, ECs produce nitric oxide (NO) which relaxes the vessel, whereas VSMCs contract in response to neurohumoral factors such as noradrenaline (NAd) and angiotensin (Ang) II [3]. Intracellular changes in Ca^2+^ concentration and Ca^2+^ signaling play a key role in regulating vascular diameter via ECs and VSMCs [4, 5].

**Fig. 1:**
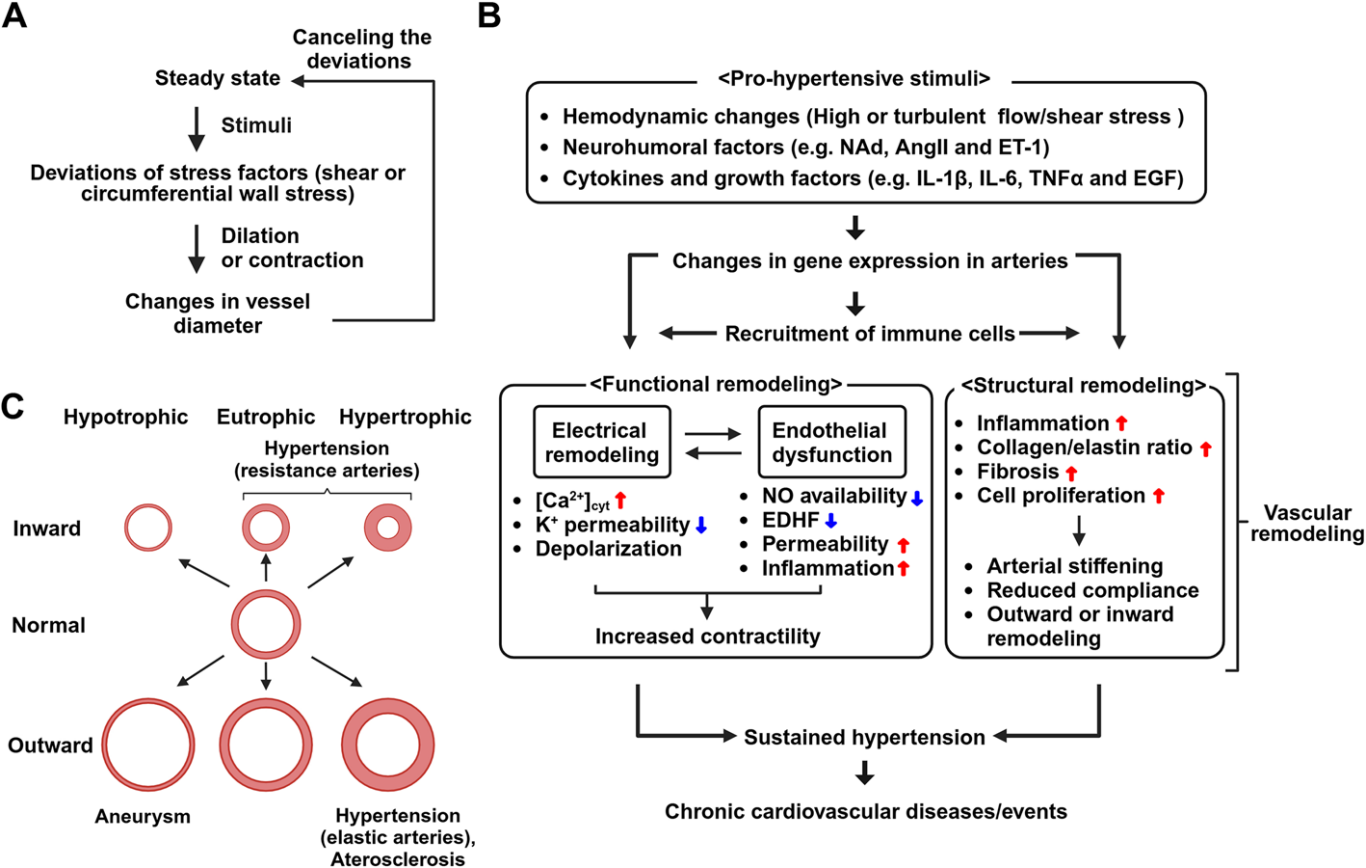
Progression and Classification of Vascular Remodeling. **A** When a stimulus is applied to an artery, stress factors (shear stress and circumferential wall stress) deviate from their normal steady state. The artery compensates for this perturbation by changing the vessel diameter through relaxation or contraction. If the stimulus is short-term, the change in vessel diameter is also temporary. **B** Conversely, if pro-hypertensive stimuli persists for a prolonged period, vascular component cells exhibit dramatic changes in transcription profiles, including ion channels that regulate intracellular Ca^2+^ signaling. This can result in electrical remodeling causing enhanced vasocontractility. At the same time, vasodilator capacity and endothelial barrier integrity are impaired. These changes cause functional remodeling. In addition, macrophages traffic to, and accumulate in the adventitia of blood vessels where they promote structural remodeling that enhances arterial stiffness and changes vessel diameter. In combination, these functional and structural remodeling lead to chronic hypertension. **C** Structural remodeling is classified on the basis of changes in vessel diameter and cross-sectional area. In hypertension, outward hypertrophic remodeling is observed in elastic arteries, whereas inward eutrophic remodeling or inward hypertrophic remodeling is observed in resistance arteries. In atherosclerosis, outward hypertrophic remodeling is observed in large vessels. EDHF: endothelium-derived hyperpolarizing factor, EGF: epidermal growth factor, ET-1: endothelin-1, NAd: noradrenaline

It is now known that the steady state of blood vessels is regulated by shear stress due to blood flow and/or circumferential wall stress. When arteries are subjected to biochemical (e.g. neurohumoral factors) or biomechanical stimuli (e.g. high blood flow and pressure overload), these stress factors change, leading to deviation from the steady state [1]. Both VSMCs and ECs act as effectors to alter the diameter of the blood vessels and thus counteract these changes (Fig. 1A). If these stimuli are brief, the changes in vessel diameter are reversible. However, if pro-hypertensive stimuli continue for a prolonged time, blood vessels undergo functional and structural changes that are termed “vascular remodeling” (Fig. 1B) [6]. Initially, vascular remodeling functions as a compensatory mechanism, but over time, it leads to the disruption of homeostasis and the development of pathological conditions. Hypertension and atherosclerosis are examples of disorders caused by vascular remodeling. These progressive conditions are major causes of cardiovascular diseases, including ischemic heart disease and stroke, which rank as the first and second leading causes of death worldwide in 2020, according to a WHO report. Therefore, understanding the mechanisms underlying these pathologies is important for developing therapeutic agents that prevent cardiovascular events. In this review, we outline the mechanisms responsible for the functional remodeling that underlies increased contractility of blood vessels, and then describe structural remodeling associated with hypertension with a focus on the relationships between vascular component cells and leukocytes.

### Process of vascular remodeling in hypertension

In this review, we will describe vascular remodeling separately as “functional remodeling” and “structural remodeling”, respectively. Persistent hypertension-inducing stimuli largely alter the expression levels and activity of ion channels in VSMCs and ECs [7]. This pattern of change is called “electrical remodeling”. It is one of the main molecular components of functional remodeling that can result in increased vasocontractility [8, 9]. At the same time, there is endothelial dysfunction which is characterized by reduced endothelium-derived relaxation, inflammatory responses, and increased endothelial permeability. In addition, during process of structural remodeling, subsets of vascular component cells can produce chemokines and adhesion molecules, which then recruit specific types of immune cells, mainly macrophages, to the vessel wall. This may promote structural changes in blood vessels [1] (Fig. 1B). It is now known that in the vessel wall, macrophages produce matrix-degrading enzymes that promote the breakdown of the extracellular matrix (ECM) [10]. Moreover, both VSMCs and fibroblasts continuously produce fibrous collagen, resulting in increased vascular fibrosis and stiffness due to fragmentation of elastin and deposition of collagen and fibronectin [11]. This promotes chronic changes in vessel diameter due to structural remodeling [1] (Fig. 1B).

Structural remodeling is classified on the basis of changes in vessel diameter and cross-sectional area [12] (Fig. 1C). An increase in vessel diameter is denoted outward remodeling, whereas a decrease in diameter is called inward remodeling. If the vascular wall area increases or decreases, this is referred to as hypertrophic or hypotrophic remodeling, respectively; whereas no significant change in wall area is referred to as eutrophic remodeling. The phenotype of this remodeling is determined by the direction of pressure applied to the vessel and the contractility of VSMCs. In hypertension, when the pressure on elastic arteries (such as the aorta) persistently increases, there is an increase in circumferential wall stress, and consequently the aorta undergoes outward remodeling with ECM reconstruction in a dilated state (Fig. 1C). In this process, the proliferation of VSMCs and fibroblasts and ECM production from these cells increase the total wall thickness and stiffness, thereby reducing the circumferential wall stress [13] (Fig. 1C). Moreover, the stiffening of elastic arteries can increase the pulse wave velocity with each heart beat; this may result in damage to resistance vessels and microvessels in peripheral organs [14]. On the other hand, when the pressure on resistance vessels persistently increases during hypertension, the lumen radius decreases due to myogenic contraction to reduce circumferential wall stress. When the ECM stiffens under these conditions, resistance vessels undergo inward remodeling [1]. In resistance arteries that have been affected by essential hypertension, inward eutrophic remodeling characterized by VSMC rearrangement and ECM accumulation is observed. In contrast, in secondary hypertension, diabetes, and salt-sensitive hypertension, inward hypertrophic remodeling is promoted by severe endothelial damage, leading to VSMC proliferation [14] (Fig. 1C). Inward remodeling is thought to be a compensatory mechanism aimed at reducing wall stress and maintaining capillary bed pressures and flow within normal ranges. However, in the long term, it reduces the blood supply to peripheral organs, leading to end-organ damage [14]. There is a positive correlation between the remodeling of elastic arteries and resistance vessels, which are both critically involved in the progression of hypertensive pathology [15]. In summary, these functional and structural remodeling events initially manifest as adaptive responses to hypertensive stimuli. In contrast, over a prolonged periods of time these changes become maladaptive due to increased vascular tone and chronic arterial stiffening and narrowing, often leading to cardiovascular diseases such as essential and secondary hypertension, atherosclerosis, and aortic dissection (Fig. 1B).

## Regulation of vascular tone by Ca^2+^ signaling in VSMCs and ECs

**Fig. 2:**
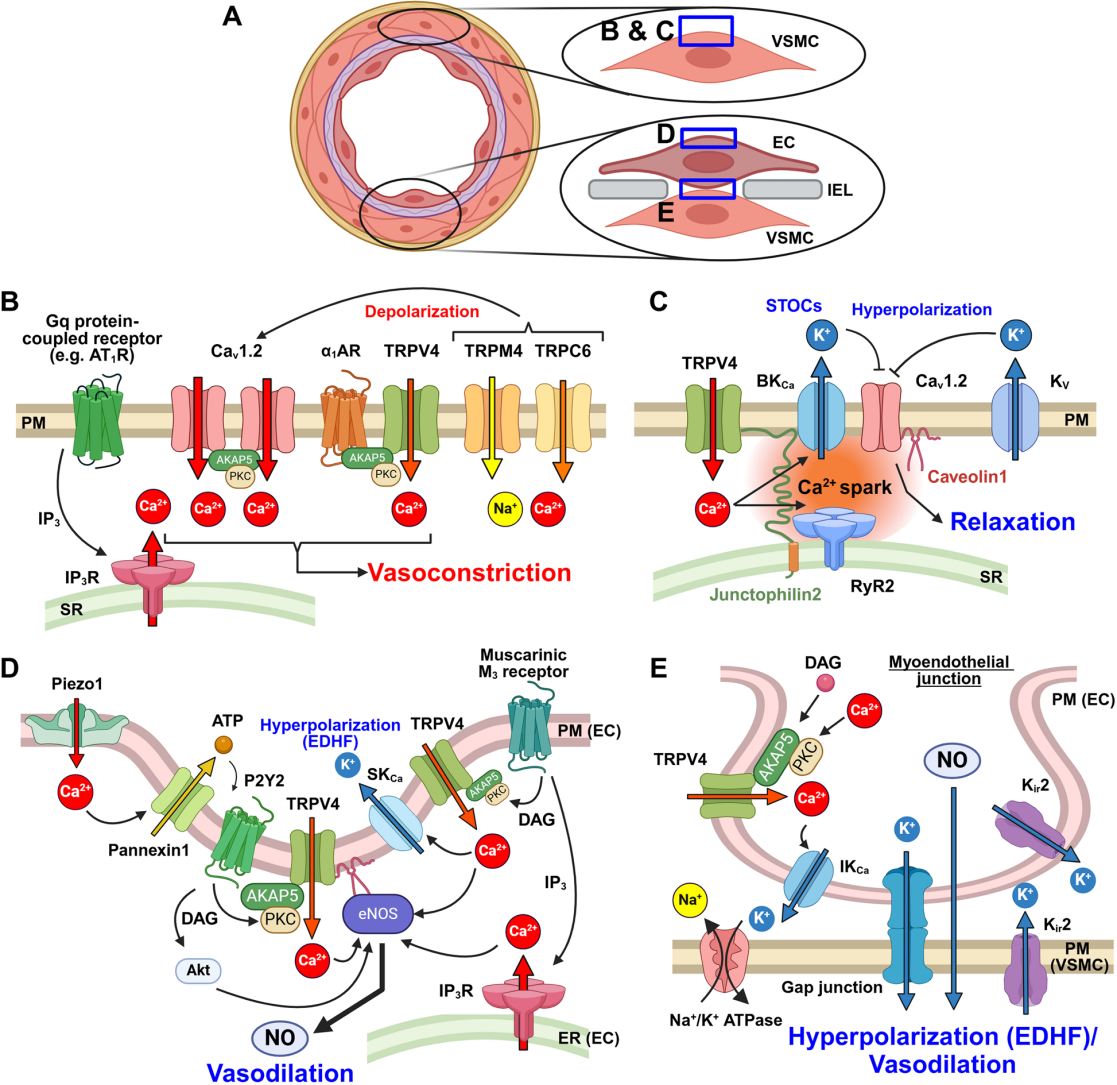
Ca^2+^ Signaling in Healthy Vascular Smooth Muscle Cells (VSMCs) and Endothelial Cells (ECs). **A** Diagrammatic illustration of the structure of blood vessels. ECs form myoendothelial junctions with VSMCs through holes/slits in the internal elastic lamina (IEL). The blue squares marked **B-E** in this figure define areas which are emphasized in the following panel (**B to E**). **B** Ca^2+^ channels involved in VSMC contraction. Ca_v_1.2 channel functions as the main Ca^2+^-permeable channel. Stretch-sensitive ion channels, TRPM4/TRPC6, can activate Ca_v_1.2 channels. TRPV4 channels are activated downstream of α_1_AR. Gq protein-coupled receptors produce IP_3_, which activates IP_3_R, and causes an increase in [Ca^2+^]_cyt._ **C** Ca^2+^ sparks activate nearby BK_Ca_ channels to induce STOCs and hyperpolarize the membrane potential. Caveolin1 and junctophilin2 are expressed in close proximity to RyR2 and BK_Ca_ channels. TRPV4 channels increase STOCs either by supplying Ca^2+^ directly to BK_Ca_ channels, or indirectly via Ca^2+^ sparks. K_v_ channels can hyperpolarize the membrane potential and suppress the activity of Ca_v_1.2 channels. **D** ACh activates IP_3_R or TRPV4 channels and increases [Ca^2+^]_cyt_, resulting in NO and EDHF production. Physiological shear stress also activates TRPV4 channels to promote NO production. Stretch or shear sensitive Piezo1 channels activate TRPV4 channels via pannexin1/P2Y2 to promote NO production. Caveolae are important for NO and EDHF production by TRPV4 channels. **E** At the myoendothelial junction, Ca^2+^ influx through TRPV4 channels activates IK_Ca_ channels and eNOS to produce EDHF and NO, respectively. K_ir_2.1 channels amplify the hyperpolarization response by IK_Ca_/SK_Ca_ channels. Gap junctions formed by connexin 37/40/43 transmit hyperpolarization to VSMCs. VSMCs are also hyperpolarized due to active K^+^ efflux mediated by Na^+^/K^+^-ATPase and K_ir_2.1 channels. AT_1_R: AngII receptor type 1, DAG: diacylglycerol, EDHF: endothelium-derived hyperpolarizing factor, ER: endoplasmic reticulum, PM: plasma membrane, SR: sarcoplasmic reticulum, STOC: spontaneous transient outward current, α_1_AR: α_1_ adrenergic receptor

Vascular diameter is controlled by the contraction and relaxation of VSMCs. ECs cover the lumen of blood vessels and contact VSMCs through holes in the internal elastic lamina (IEL) (Fig. 2A). This structure is called the myoendothelial junction where ions and small molecules can pass between VSMCs and ECs through gap junctions [16]. In this section, we will describe the ion channels that play important roles in Ca^2+^ signaling-mediated regulation of vascular tone.

### Ca^2+^ signaling in VSMCs in healthy arteries

VSMC contraction is promoted by stimuli such as sympathetic nerve activation, vasoconstrictors such as AngII and endothelin (ET)-1, and pressure overload. All of these stimuli increase cytosolic Ca^2+^ concentration ([Ca^2+^]_cyt_), which causes VSMC contraction through Ca^2+^/Calmodulin (CaM)-mediated activation of myosin light chain kinase and phosphorylation of myosin light chain [2]. When VSMCs are depolarized, [Ca^2+^]_cyt_ increases mainly due to Ca^2^⁺ influx via L-type voltage-dependent Ca^2^⁺ (Ca_v_1.2) channels [17] (Fig. 2B). Mechanical stretch also activates mechanosensitive cation channels such as, TRPC6 [18] and TRPM4 [19] depolarizing the membrane potential and opening Ca_v_1.2 channels. Inositol 1,4,5-trisphosphate receptors (IP_3_R) in the sarcoplasmic reticulum (SR) release Ca^2^⁺ into the cytosol and cause vasoconstriction [20] (Fig. 2B). In contrast, ryanodine receptors (RyR) cause local Ca^2+^ release called Ca^2^⁺ sparks [21] that activate nearby large-conductance Ca^2^⁺-activated K⁺ (BK_Ca_) channels to induce spontaneous transient outward currents (STOCs) [22] (Fig. 2C). This hyperpolarizes the membrane potential, closing L-type Ca^2^⁺ channels and inhibiting Ca^2^⁺ influx, resulting in vasodilation. We previously found that BK_Ca_ channels are localized to caveolae by binding to caveolin1, a caveolar forming protein, in VSMCs, and this promoted the frequency of STOCs [23]. In addition, junctophilin2 brings BK_Ca_ channels in caveolae and RyR on the SR into close proximity, which increases the efficiency of Ca^2+^ spark-STOC coupling and reduces the tension of mesenteric arteries [24]. In addition to BK_Ca_ channels, the voltage-dependent K^+^ (K_v_) channel family (K_v_1 [25], K_v_2 [26], and K_v_7 [27]) counteracts vasocontraction by suppressing Ca^2+^ influx from Ca_v_1.2 channels by hyperpolarizing the membrane potentials (Fig. 2C).

TRPV4 is a cation channel that is activated by stretch, arachidonic acid, and temperature, and has attracted attention in vascular biology research recently. In VSMCs, TRPV4 channels form a molecular complex with RyR and BK_Ca_ channels [28]. This increases the Ca^2+^ spark activity by activating RyR, and increases STOCs by directly supplying Ca^2+^ to BK_Ca_ channels, thus decreasing vascular tone [28, 29] (Fig. 2C). TRPV4 channels also form a molecular complex with α_1_ adrenergic receptors (α_1_AR) and protein kinase C (PKC) via A-kinase anchoring protein (AKAP) 150. PKC activates TRPV4 channels, enhancing VSMC contraction induced by sympathetic nerve stimulation [29] (Fig. 2B). Super-resolution microscopy has revealed that the above two molecular complexes are localized in separate compartments on the plasma membrane of VSMCs [29]. Comparative analysis of EC-specific and SMC-specific TRPV4-knockout mice showed that TRPV4 channels in VSMCs cause vasoconstriction induced by phenylephrine and U46619, suggesting that TRPV4 channels primarily contribute to contraction in VSMCs [30].

### Ca^2+^ signaling in ECs in healthy arteries

ECs produce NO via endothelial nitric oxide synthase (eNOS), which relaxes VSMC and dilates blood vessels. eNOS binds to caveolin1 in the steady state in an inactivated state [31]. When acetylcholine (ACh) released from parasympathetic nerve terminals binds to muscarinic ACh M_3_ receptors to increase [Ca^2+^]_cyt_, Ca^2+^/CaM binds to eNOS and dissociates from caveolin1, activating eNOS. On the other hand, in resistance vessels, vasodilation by endothelium-derived hyperpolarizing factor (EDHF) is dominant [32], contributing to local blood flow regulation. Although the actual nature of EDHF remains controversial, stimuli such as ACh and shear stress increase [Ca^2+^]_cyt_ in ECs and subsequently activate intermediate-conductance Ca^2+^-activated K^+^ (IK_Ca_) channels and small-conductance Ca^2+^-activated K^+^ (SK_Ca_) channels. This results in membrane hyperpolarization, which is transmitted to VSMCs via gap junctions in myoendothelial junction to cause VSMC relaxation [33].

TRPV4 [34] and Piezo1 [35] are ion channels that allow Ca^2+^ influx into ECs in response to ACh and shear stress. TRPV4 channels are localized in caveolae and form molecular complexes with connexin 43 and SK_Ca_3 channels to induce EDHF [36, 37] (Fig. 2D). In ECs from caveolin1 knockout mice, ACh-induced NO production is increased, while reduced TRPV4 channel activity and loss of ACh-induced EDHF are observed [36]. Laminar shear stress induces TRPV4/eNOS/caveolin1 microdomains at the downstream end of ECs, which activate eNOS through TRPV4 channel-mediated Ca^2+^ influx and suppress the expression of NF-kB-dependent inflammatory genes [38]. Piezo1 channels are localized on the luminal side of ECs or at the junction between ECs. They are activated by shear stress and are permeable to Ca^2+^. Piezo1 channels are required for fluid-directed alignment of ECs [35]. They activate eNOS by promoting ATP release via pannexin and activating the purinergic P2Y2 receptor, which phosphorylates eNOS at Ser1177 [39] (Fig. 2D).

In the myoendothelial junction, TRPV4 channels form a complex with PKC via AKAP150. ACh stimulation activates TRPV4 channels and evokes EDHF by activating IK_Ca_ channels [40] (Fig. 2E). The inwardly rectifying K^+^ (K_ir_2.1) channel amplifies this hyperpolarization [33]. An increase in the extracellular K^+^ concentration at myoendothelial junction shifts the K^+^ equilibrium potential towards more depolarized potentials, promoting K^+^ efflux via K_ir_2.1 channels, hyperpolarizing the membrane potential of ECs and VSMCs [41]. The Na^+^/K^+^ ATPase enhances EDHF by K^+^ uptake and Na^+^ release which hyperpolarizes the membrane potential [42].

## Functional remodeling of arteries in hypertension

In hypertension, peripheral vascular resistance increases continuously due to both functional and structural remodeling of systemic arteries. In both hypertensive patients and animal models, functional remodeling (characterized by decreased endothelial function, increased vascular contractility, and increased responsiveness to agonists and mechanical stimuli) are commonly observed [7, 15]. Electrical remodeling due to changes in the expression and activity of Ca^2+^ channels or K^+^ channels underlies functional remodeling [8, 9] (Fig. 1B). Below, we discuss recent findings on electrical/functional remodeling.

### Electrical remodeling in hypertensive VSMCs

**Fig. 3:**
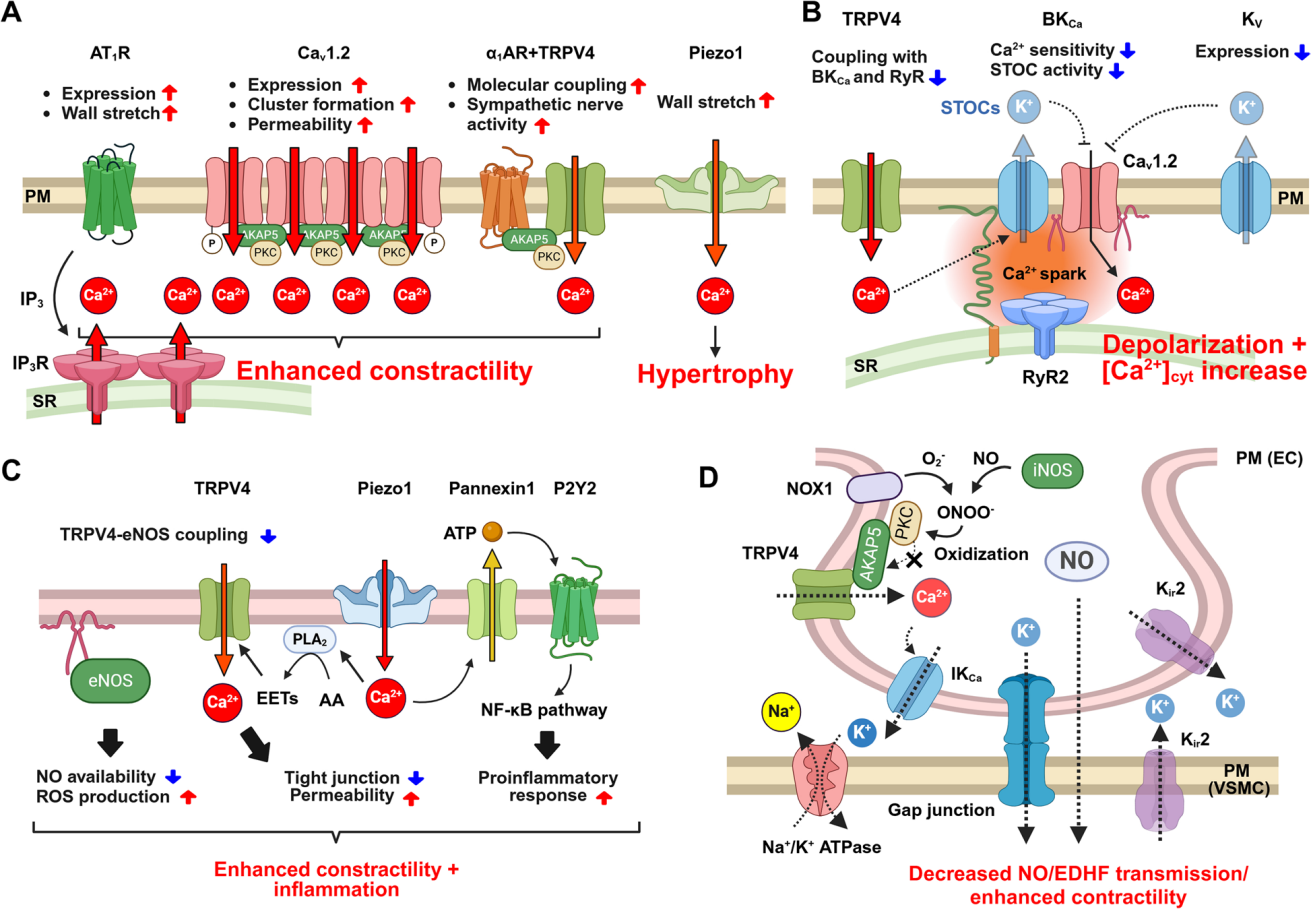
Electrical remodeling in hypertensive VSMCs and ECs. **A** In hypertensive VSMCs, the expression and activity (cluster formation) of Ca_v_1.2 channels are enhanced. In addition, increased expression of AT_1_R and IP_3_R and increased formation of the α_1_AR-TRPV4 complexes can increase VSMC contractility. **B** Decreased activity of BK_Ca_ channels and dissociation of TRPV4 channel reduce STOCs. K_v_ channel expression also decreases, resulting in depolarization of the membrane potential. **C** In ECs, dissociation of TRPV4 channel and eNOS reduces NO production. Activation of Piezo1 channels can cause sustained Ca^2+^ influx from TRPV4 channels due to sustained high shear stress, enhancing endothelial permeability. In addition, Piezo1 channels activate a NF-κB pathway mediated by panexin1/P2Y2 in response to turbulent laminar flow. **D** In myoendothelial junctions, downregulation or oxidation of AKAP150 reduce TRPV4 channel activity and reduce EDHF and NO production. K_ir_2.1 channel and gap junction component proteins are also downregulated in hypertensive ECs and VSMCs localized at the myoendothelial junction. AA: arachidonic acid, AT_1_R: AngII receptor type 1, EDHF: endothelium-derived hyperpolarizing factor, EET: epoxytrienoic acid, PLA_2_: phospholipase A_2_, PM: plasma membrane, SR: sarcoplasmic reticulum, STOC: spontaneous transient outward current, α_1_AR: α_1_ adrenergic receptor

It has been reported that continuous pressure overload for two days on arteries increases the expression of Ca_v_1.2 channels and depolarizes the membrane potential (from −50 mV to −40 mV) [43] (Fig. 3A). In this study, the expression of Ca_v_1.2 channels increased simply by culturing arteries in high KCl medium, suggesting that continuous membrane depolarization maintains the increased expression of Ca_v_1.2. ET-1 induces reactive oxygen species (ROS) production from mitochondria via Ca^2+^ release from IP_3_R and promotes Ca_v_1.2 transcription via the NF-kB pathway [44]. Mineralocorticoid receptors (MR) in VSMCs increase Ca_v_1.2 channel and AngII receptor (AT_1_R) expression, AngII-induced ROS production, and vascular tone by downregulating miR155 in an age-dependent hypertension model [45]. During hypertension, not only the expression level of Ca_v_1.2 channels but also its Ca^2+^ permeability increases. A “Ca^2+^ sparklet” is a very small, localized and transient influx of Ca^2+^ mainly through Ca_v_1.2 channels. In VSMCs, Ca_v_1.2 clusters form molecular complexes with AKAP150 and PKCα [46]. AngII promotes PKC-mediated phosphorylation of Ca_v_1.2 channels at Ser1928, increases the number of Ca_v_1.2 channels in the clusters, thereby promoting Ca^2+^ sparklets [47]. (Fig. 3A). This cascade results in increased [Ca^2+^]_cyt_ and enhanced vasoconstriction. At the same time, the calcineurin-NFAT system is also activated, and the transcription and expression of K_v_2.1 channels [48] and the BK_Ca_ channel β1 subunit [49] are reduced, depolarizing the membrane potential and maintaining the hypertensive state. This increase in Ca^2+^ sparklets is dependent on the scaffolding protein AKAP150, since knockout of AKAP150 blunted AngII-induced hypertension [46]. In spontaneously hypertensive rats (SHRs), upregulation of AKAP150 increases Ca^2+^ sparklet activity mediated by PKC. Interestingly, it has been reported that exercise reduces the expression of AKAP150 in SHRs, which normalizes Ca^2+^ sparklet activity and blood AngII concentration, and blood pressure [50].

TRPV4 channels are involved in both the contraction response mediated by the AKAP150/α_1_AR/PKC molecular complex and the vascular relaxation response mediated by the RyR/BK_Ca_ channel molecular complex [29]. In VSMCs from hypertensive patients and AngII-induced hypertension models, the TRPV4/AKAP150/α_1_AR/PKC molecular complex was increased, enhancing the contribution of TRPV4 channels to vasoconstriction (Fig. 3A), whereas the TRPV4/RyR/BK_Ca_ channel molecular complex was decreased (Fig. 3B) [29]. Similar results were observed in obesity-induced hypertension, suggesting that TRPV4 channel activity contributes to increased tone, elevated blood pressure, and medial thickening during hypertension [30]. Piezo1 channels are highly expressed in VSMCs of resistance vessels and depolarize the membrane potential in response to mechanical stimulation, whereas they are not involved in myogenic tone [51]. In AngII- or DOCA/salt-induced hypertension models, Piezo1 contributes to medial thickening by increasing transglutaminase expression (Fig. 3A). In addition, increased expression of AT_1_R [45] and IP_3_R [52] also contribute to an increase in VSMC contractility. Since AT_1_R can be activated by pressure overload alone in an AngII independent manner [53], both increased expression and high intraluminal pressure may synergistically promote vasocontractility in VSMCs.

Ca^2+^ spark-STOC coupling is important for maintaining tone, but it is known that this coupling is impaired in hypertensive conditions (Fig. 3B). It has been proposed that genetic deletion [54] or reduced expression [55] of the BK_Ca_ channel β1 subunit reduces Ca^2+^ sensitivity, making it impossible to induce STOCs in response to Ca^2+^ sparks. In BPH/2 mice, dissociation of the plasma membrane and SR occurs in cerebral resistance arteries, and Ca^2+^ spark-STOC coupling is weakened, leading to reduced cerebral blood flow and vascular dementia [56]. In addition, the expression of K_v_ channels such as K_v_1 [25], K_v_2.1 [26], and K_v_7 [27] is reduced, which is thought to depolarize the membrane potential. The synergistic effect of increased expression/activity of Ca_v_1.2 channels and depolarization increase [Ca^2+^]_cyt_, enhancing vasocontraction.

### Electrical remodeling in hypertensive ECs

Healthy ECs suppress excessive contraction of arteries by NO or EDHF, but in hypertensive conditions, pressure overload, turbulent flow, and humoral factors such as AngII and ET-1 cause superoxide production via NADPH oxidase. Superoxide reduces the bioavailability of NO by uncoupling eNOS and converting NO to ONOO^−^, thereby attenuating vasorelaxation and increasing peripheral vascular resistance [57]. In AngII-induced hypertension mice, the molecular coupling between TRPV4 channels and eNOS in aortic ECs is reduced, resulting in decreased NO production [58] (Fig. 3C). The turbulent flow leads to abnormal activation of Piezo1 channels, which activates the pannexin1-P2Y2-PLC-α5 integrin-FAK pathway [59]. FAK induces endothelial activation and transcription of pro-inflammatory factors via the NF-kB pathway (Fig. 3C). Piezo1 channels also activate TRPV4 channels via phospholipase A_2_, causing a sustained increase in [Ca^2+^]_cyt_ [60]. This leads to the disassembly of adherent junctions between ECs and increased endothelial permeability. These findings suggest that the state of blood flow may alter the signal transduction initiated by Piezo1 and TRPV4 channels by changing the components of molecular complexes containing these channels. Thus, during abnormal blood flow, Piezo1 and TRPV4 channels promote leukocyte adhesion to ECs and infiltration into the subendothelial space, leading to inflammation in the vascular wall.

At the myoendothelial junction of AngII-induced hypertension mice, AKAP150 expression is attenuated, and ACh-induced EDHF generation by the TRPV4/AKAP150/PKC complex is reduced [40] (Fig. 3D). Importantly, the density of myoendothelial junctions is reduced and their local IP_3_-stimulated Ca^2+^ signals are blunted in mesenteric arterioles of SHRs [61]. In an obesity-induced hypertension model, NOX1 and iNOS expression are increased at the myoendothelial junction of ECs, converting NO to ONOO^−^ [62]. ONOO^−^ oxidizes Cys36 of AKAP150, dissociating PKC, and attenuating TRPV4 channel activity and thus EDHF.

In a hypertensive vascular dementia model based on BPH/2 mice, blood supply to metabolically active areas of the brain (functional hyperemia) was reduced through reduced activity of K_ir_2.1 channels in cerebral microvascular ECs [63]. Interestingly, amlodipine, but not losartan, inhibited this functional reduction in hyperemia. This difference is blunted by additional treatment with eplerenone, indicating pro-dementia roles of aldosterone. This suggests that K_ir_2.1 channels may be a target for preventing cognitive decline in late life in hypertensive patients treated with AT_1_R blockers.

## Structural remodeling of arteries in hypertension

In hypertension, structural remodeling (mainly outward remodeling of elastic vessels and inward remodeling of resistance vessels) occurs due to long-term pro-hypertensive stimuli (Fig. 1B). VSMC proliferation is suppressed at steady state and in the early stage of hypertension, but as hypertension progresses, VSMCs enter the cell cycle and begin to proliferate [64]. In this process, Ca^2+^ signals are important (for review, see [65–68]). Previous studies have revealed that leukocytes such as macrophages accumulating in the vascular wall promote turnover of the ECM and cause VSMC migration and proliferation, which are very important for the initiation and progression of structural remodeling in various vascular diseases including hypertension. In this section, we will first outline the role of immune cells in hypertension. Next, we will summarize research findings that have clarified the molecular mechanism by which blood flow disorders and pressure overload cause leukocyte accumulation in the vascular wall based on data sets obtained from in vivo and ex vivo experimental systems.

### Immune cells relating to structural remodeling

**Fig. 4:**
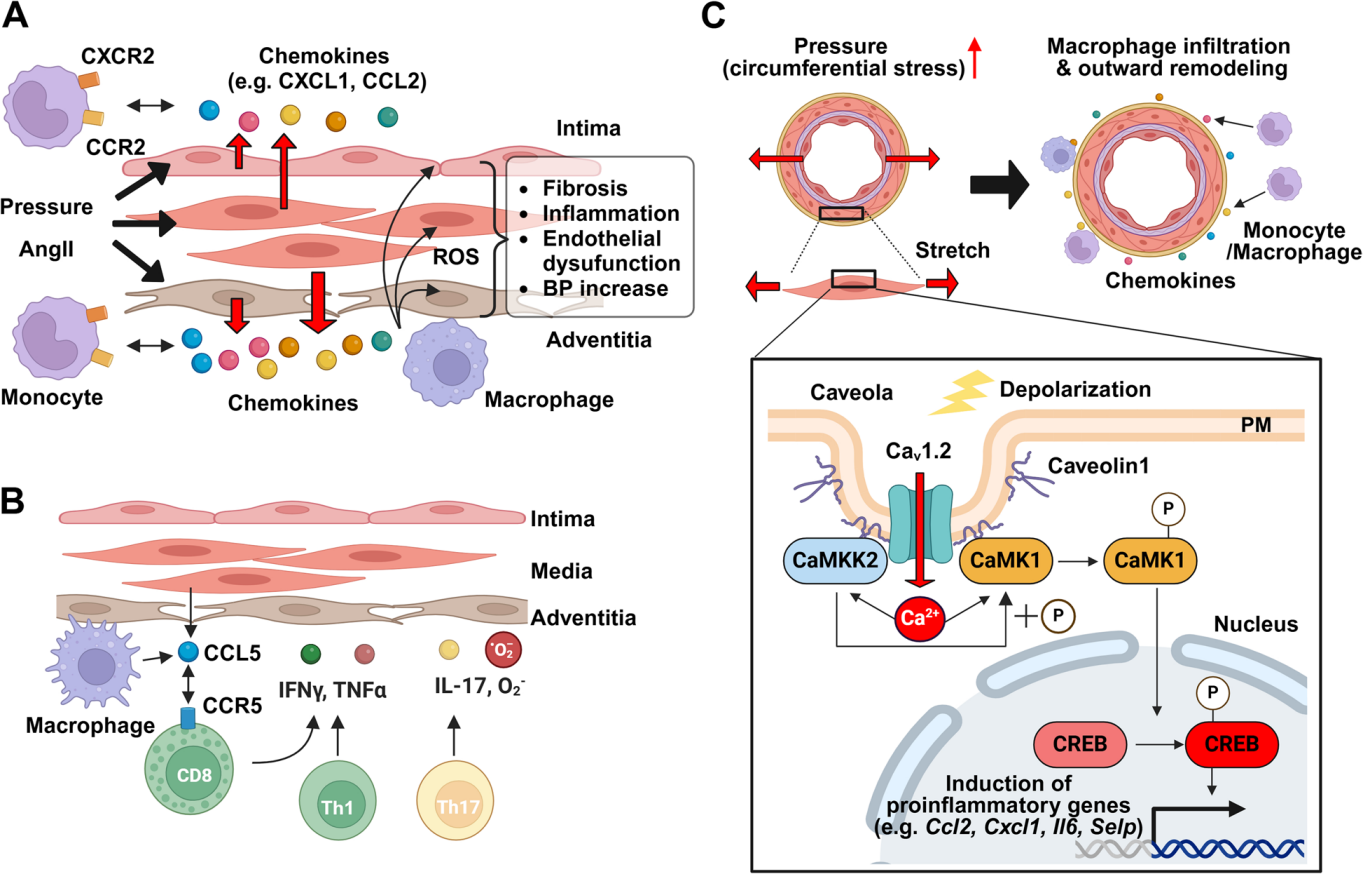
Structural Remodeling mediated by Interactions between Vascular Component and Immune Cells. **A** Pressure load changes on blood vessels and/or Ang II initiate or promote the production of chemokines from ECs, VSMCs, and adventitial fibroblasts, leading to monocyte and macrophage infiltration into the vessel wall. ROS produced by macrophages and vascular component cells cause vascular damage. **B** CD8^+^ T cells and probably Th1 cells accumulate in the vessel wall through CCL5-CCR5 interactions, where they produce IFNγ and TNFα. In addition, Th17 cells produce IL-17 and superoxide. These cytokines and ROS both can promote hypertension. **C** Elevated local blood flow in the mesenteric artery increases circumferential stress in the vascular wall by stretching VSMCs and depolarizing the membrane potential. The scaffolding protein caveolin1 mediates the formation of a molecular complex consisting of Ca_v_1.2 channels, CaMKK2, and CaMK1. Ca^2+^ influx through Ca_v_1.2 channels can activate CaMKK2 and CaMK1, and this causes CaMK1 to translocate to the nucleus where it induces the transcription of genes that encodes chemokines and adhesion molecules such as CXCL1, CCL2, P-selectin, and VCAM1. As a result, monocytes and macrophages accumulate in the adventitia, leading to outward remodeling. BP: blood pressure, CaMKK2: Ca^2+^/CaM dependent kinase kinase2, PM: plasma membrane

Experiments using C-C motif chemokine receptor (CCR) 2 knockout mice [69] and LysM-iDTR mice (in which monocytes are depleted) [70] have revealed that monocytes and macrophages play crucial roles in the development of hypertension. The macrophages that accumulate in the adventitia in response to AngII initially exhibit M1-like (classically activated) properties but shift to M2-like (alternatively activated) macrophages after 14 days, leading to chronic hypertension through collagen deposition and elastin degradation [71] (Fig. 4A). Mechanical stress on blood vessels and Ang II stimulation increase the expression of CCL2 in ECs [72], VSMCs [73], and adventitious fibroblasts [74], leading to monocyte accumulation in the vascular wall and differentiation into macrophages (Fig. 4A). In addition, during hypertension, the expression of C-X-C motif chemokine ligand (CXCL) 1 in the aorta and CXCR2 in monocytes/macrophages increases, leading to increased macrophage accumulation [75]. Accumulated macrophages produce ROS and cytokines, leading to further ROS production by VSMCs and ECs and resulting in elevated blood pressure, aortic fibrosis, inflammation, and endothelial dysfunction.

For over 20 years, it has been known that T cells accumulate around blood vessels and in the kidneys in the setting of progressive hypertension and that immunosuppressants lower blood pressure [76, 77] (Fig. 4A). To date, it has been revealed that among T cell subsets, interferon (IFN) γ-producing CD8^+^ cells are involved in blood pressure elevation and vascular rarefaction in the kidneys [78]. Moreover, CCL5-CCR5 is involved in the infiltration of T cells into the vascular wall [79] (Fig. 4B). CD4^+^-derived Th17 cells produce superoxide via interleukin (IL)-17A, contributing to blood pressure elevation, endothelial dysfunction, fibrosis, and sodium reabsorption in AngII-induced hypertension [80]. In hypertension, efferent sympathetic nerve activity increases [3], and these immune cells are activated in secondary lymphoid tissues such as the spleen and lymph nodes and in the kidney, and then distributed throughout the body, enhancing inflammation [81].

### Pressure-induced structural remodeling

Either pressure overload (150 mmHg) or AngII treatment can result in the production of transforming growth factor α and transactivation of epidermal growth factor receptor (EGFR) secondary to ROS production by NADPH oxidase in mouse carotid arteries [82]. This actives the NF-kB pathway, promoting survival of vascular cells, upregulating CCL2, CXCL1, IL-6, and vascular cell adhesion molecule (VCAM) 1, and increasing monocyte accumulation in the vascular wall, and thus resulting in outward remodeling [83]. Importantly, ONOO^−^-induced matrix metalloproteinase (MMP) 9 expression is required for this outward remodeling [84]. A similar function of MMP9 is also observed in outward remodeling that occurs in the model of early stages of atherosclerosis [85] and vascular injury following carotid artery ligation [86]. MMP9 expression is increased in elastic vessels in the early stages of hypertension, and it is thought that the compensatory outward remodeling contributes to the suppression of systolic blood pressure rise, the reduction of pulse pressure, and the homeostatic maintenance of vessel compliance.

In contrast to these ROS-mediated pathways, we have found that Ca^2+^ signaling induced by mechanical stress applied to blood vessels is converted into gene transcription via excitation-transcription (E-T) coupling [48, 67], leading to vascular remodeling (Fig. 4C). In the mesenteric artery ligation model, we showed that increased circumferential wall stress accompanying increases blood flow in the mesenteric artery activates Ca_v_1.2 channels in VSMCs and an associated increase in Ca^2+^ influx into the cytoplasm. This then activates Ca^2+^/CaM dependent kinase kinase (CaMKK) 2 and CaMK1, which forms a complex with Ca_v_1.2 channels via the caveolin1 [23], and CaMK1 translocates to the nucleus, where it activates the transcription factor CREB [87]. The aggregate result is an upregulation of the expression of genes encoding chemokines and adhesion molecules such as CXCL1, CCL2, P-selectin, and VCAM1, again resulting in macrophage accumulation in the adventitia and outward remodeling [88]. Macrophage accumulation and outward remodeling due to the increased circumferential wall stress are attenuated in caveolin1 knockout mice or by administration of the CaMKK2 inhibitor STO609. These findings suggest that VSMCs can produce inflammatory factors such as chemokines, which attract monocytes and macrophages to the vessel wall (mainly in the adventitia).

In atherosclerosis, monocytes, macrophages, and T cells primarily infiltrate from the luminal side of blood vessels. In contrast, it has been shown that in hypertension, monocytes, macrophages, and T cells accumulate on the adventitia side of blood vessels. Similar trends are also seen in aortic dissection [89] and pulmonary hypertension [90]. Although the ability of VSMCs to produce inflammatory cytokines is weaker than that of immune cells, the large number of VSMCs in blood vessels compared with other cell types suggests that the inflammatory response of VSMCs may be sufficient to trigger subsequent immune responses [91]. While VSMCs are not in direct contact with either the vessel lumen or extravascular regions, arteries are permeable to low-molecular-weight compounds and albumin (66 kDa) [92]. In particular, since the adventitia lacks physical barriers such as the tight junctions within the endothelium, chemokines (8–12 kDa [93]) produced by VSMCs are likely to diffuse relatively freely within the vessel, thereby promoting the infiltration of monocytes and macrophages into the adventitia.

It is now well established that caveolin1 is an aggravating factor for hypertension and atherosclerosis. In an AngII-induced hypertension model created in caveolin1 knockout mice, no difference was observed in systemic blood pressure, but phosphorylation of EGFR, upregulation of caveolin1 and adventitial VCAM1, medial thickening, and fibrosis were suppressed in the aorta, coronary artery, and renal artery [94]. Plaque formation was also suppressed in caveolin1 and eNOS double knockout mice, revealing that the protective effect of caveolin1 deficiency is not related to NO [95]. Caveolin1 deficiency suppressed LDL transport through ECs, fibronectin accumulation, inflammatory responses, and macrophage infiltration. Furthermore, the distribution of caveolae showed a different pattern in areas exhibiting plaque formation compared with those that did not. Both activation of the NLRP3 inflammasome and production of IL-1β in macrophages are important for the development of hypertension [96]. When we investigated the role of caveolin1 in macrophages, we unexpectedly found that lipopolysaccharide-induced transcription of pro-inflammatory factors, purinergic P2X7 receptor activity, and IL-1β production after ATP stimulation were all increased in caveolin1 knockout macrophages [97]. This pattern of results revealed that, unlike VSMCs and ECs, caveolin1 suppresses inflammatory responses in these macrophages.

## Prospects for the development of new therapeutic drugs for vascular diseases

To date, although excellent drugs have been developed for hypertension, there are remaining clinical challenges and knowledge gaps called a “residual risk” in hypertension [98]. It has been shown that the risk for significant cardiovascular complications (e.g. coronary disease, stroke, and cardiovascular death) of well-treated and controlled hypertensive subjects is higher than that of untreated normotensive subjects at the same blood pressure level [99]. Recently, the CANTOS (Canakinumab Anti-Inflammatory Thrombosis Outcomes Study) trial demonstrated that the IL-1β antagonist canakinumab significantly suppressed the occurrence of adverse cardiovascular events such as myocardial infarction, stroke, and cardiovascular death [100]. In addition, it has been reported that anti-inflammatory drugs such as colchicine and tumor necrosis factor inhibitors alleviate hypertension and suppress cardiovascular events in atherosclerosis [93]. In the future, it may be necessary to introduce drugs into conventional hypertension treatment that not only lower blood pressure but also suppress inflammation in blood vessels and other organs. In this regard, some of the molecules and signaling pathways discussed in this review may also become new therapeutic targets. Caveolin1 is involved in the formation of some vascular diseases, including hypertension [94], atherosclerosis [95], and pulmonary hypertension [101]. The membrane-permeable peptide “cavtratin” that mimics the functions of caveolin1 shows therapeutic effects on some cancers [102] and an intraocular angiogenesis [103] due to the inhibition of angiogenesis. In contrast, “cavnoxin” has also been developed as a competitive peptide of caveolin1. Its antihypertensive effects are due to increasing the activity of eNOS [104]. In addition, a specific inhibitor of Ca_v_1.2 channels localized in caveolae has been developed [105]. Unfortunately, however, it turned out that the inhibitor did not have a preventive effect on heart failure [106]. Thus, it may now be possible to specifically control the function of target molecules that are localized in caveolae. This capability enables lowering [Ca^2+^]_cyt_, increasing NO production, and preventing inflammatory responses and structural remodeling. The scaffolding molecule AKAP150 (AKAP5 in human) (Fig. 3) is also associated with hypertension [40, 46, 62]. Indeed, it has been shown that exercise can reduce AKAP150 in VSMCs and lower blood pressure [50]. Therefore, AKAP5 may be a promising target for hypertension by specifically modulating hypertensive signaling pathways.

## Conclusion

Ca^2+^ signaling in both VSMCs and ECs plays critical roles in maintaining vascular homeostasis. Sustained hypertensive stress on blood vessels induces both functional and structural remodeling, at least in part, by modifying Ca^2+^-dependent pathways. Thus, hypertension develops through the cooperation of functional remodeling (enhanced contractility) and structural remodeling (arterial stiffening). It is known that hypertension can be caused by abnormalities not only in blood vessels but also in other organs such as the central nervous system, sympathetic nervous system, immune system, and/or the kidneys. As a consequence, in addition to research focusing on the molecular complexes in vascular constituent cells, it will be necessary to reveal the significance of these molecular complexes at organ, inter-organ, and whole body levels to achieve optimal development and validation of new antihypertensive drugs that may prevent adverse cardiovascular events.

## Data Availability

Not applicable.

## Abbreviations

Ang: Angiotensin
AT_1_R: AngII receptor type 1
EC: Endothelial cell
eNOS: Endothelial nitric oxide synthase
EDHF: Endothelium-derived hyperpolarizing factor
ECM: Extracellular matrix
IP_3_R: Inositol 1,4,5-trisphosphate receptor
IK_Ca_ channel: Intermediate-conductance Ca^2+^-activated K^+^ channel
IEL: Internal elastic lamina
[Ca^2+^]_cyt_: Cytosolic Ca^2+^ concentration
K_ir _ channel: Inwardly rectifying K^+^ channel
BK_Ca _ channel: Large-conductance Ca^2+^-activated K^+^ channel
MMP: Matrix metalloproteinase
NLRP: NLR family pyrin containing
ROS: Reactive oxygen species
RyR: Ryanodine receptor
SR: Sarcoplasmic reticulum
SK_Ca _ channel: Small-conductance Ca^2+^-activated K^+^ channel
STOC: Spontaneous transient outward current
SHR: Spontaneously hypertensive rat
VSMC: Vascular smooth muscle cell
K_v _ channel: Voltage-dependent K^+^ channel
α_1_AR: α1 Adrenergic receptor
